# Biocontrol ability of *Bacillus velezensis* T9 against *Apiospora arundinis* causing Apiospora mold on sugarcane

**DOI:** 10.3389/fmicb.2023.1314887

**Published:** 2023-12-22

**Authors:** Jie Liao, Xuelian Liang, Huiling Li, Leixing Mo, Renfu Mo, Wei Chen, Yuning Wei, Tianshun Wang, Wenyan Jiang

**Affiliations:** Agro-Products Quality Safety and Testing Technology Research Institute, Guangxi Academy of Agricultural Sciences, Nanning, China

**Keywords:** *Apiospora arundinis*, *Bacillus velezensis*, sugarcane, 3-nitropropionic acid, biological control

## Abstract

Sugarcane (*Saccharum officinarum* L.) may be infected with *Apiospora*, which can produce the toxin 3-nitropropionic acid (3-NPA) during improper transportation and storage. The consumption of sugarcane that contains 3-NPA can lead to food poisoning. Therefore, this study sought to explore a novel biocontrol agent to prevent and control Apiospora mold. Bacteria were isolated from the soil of healthy sugarcane and identified as *Bacillus velezensis* T9 through colony morphological, physiological and biochemical characterization and molecular identification. The inhibitory effect of *B. velezensis* T9 on Apiospora mold on sugarcane was analyzed. Assays of the cell suspension of strain T9 and its cell-free supernatant showed that T9 had significant *in vitro* antifungal activities against *Apiospora arundinis* and thus, would be a likely antagonist. Scanning electron microscopy and transmission electron microscopy showed that treatment with T9 significantly distorted the *A. arundinis* mycelia, perforated the membrane, contracted the vesicles, and decomposed most organelles into irregular fragments. A re-isolation experiment demonstrates the ability of T9 to colonize the sugarcane stems and survive in them. This strain can produce volatile organic compounds (VOCs) that are remarkably strong inhibitors, and it can also form biofilms. Additionally, the cell-free supernatant significantly reduced the ability of *A. arundinis* to produce 3-NPA and completely inhibited its production at 10%. Therefore, strain T9 is effective at controlling *A. arundinis* and has the potential for further development as a fungal prevention agent for agricultural products.

## Introduction

Sugarcane (*Saccharum officinarum* L.) is the primary sugar crop that is cultivated in tropical and subtropical regions (Souza et al., 2019; Dhansu et al., 2023). As a fresh product, chewing cane is widely cultivated in many countries of the world due to its high contents of amino acids, vitamins, and trace elements that are essential for human survival. In China, the area planted to fresh sugarcane reaches 230,000 hm^2^ per year, with an output that exceeds 35 million tons (Wang et al., 2020). However, it is easily infected by various pathogens during transportation and storage. Ever since the first case report of poisoning due to the consumption of moldy sugarcane in northern China in 1972 (Liu et al., 1984), more cases have been recorded almost every year. *Apiospora* has been identified as the etiological fungus that causes moldy sugarcane poisoning (MSP) during improper postharvest storage (Liu et al., 1984; Hou et al., 1989; Liu et al., 1993). Notably, the toxic metabolite 3-nitropropionic acid (3-NPA) produced by *Apiospora* has been identified as the causal agent of poisoning (Hu et al., 1986). Based on the morphological characteristics, such as the shape of conidia and conidiophores, species of *Apiospora* that have been isolated from moldy poisoned sugarcane can be classified and identified as *Apiospora. sacchari, Apiospora. saccharicola* and *Apiospora. phaeospermum* (Liu et al., 1988). In our previous study, *A. arundinis* that produced 3-NPA was isolated from moldy sugarcane (Liao et al., 2022).

3-NPA is a small molecular biotoxin with a simple structure, and it primarily originates from moldy sugarcane caused by *Apiospora*. However, it can also be found in some plants infected with other fungi (Hajnal et al., 2020). Large amounts of 3-NPA are reported to be highly toxic to humans and animals and cause central nervous system (CNS) lesions. The clinical manifestations of 3-NPA include mild gastrointestinal symptoms, such as nausea, vomiting, abdominal pain, and diarrhea, that occur at approximately 2 h to 5 h after the consumption of moldy sugarcane. Some patients have vertigo, blurred vision, and an inability to stand, with trembling limbs, prophylaxis tetanus, and coma in severe cases. More people with severe cases die within 1–3 d or develop neurological sequelae that are similar to encephalitis and lose the ability to care for themselves (He et al., 1995; Nony et al., 1999; Birkelund et al., 2021). Therefore, preventing sugarcane from fungal colonization during storage or transportation has become an urgent scientific problem.

Currently, the main measures to ensure the safety and edibility of chewing cane are to observe whether the chewing cane has symptoms of mold, such as light yellow brown, dark brown, or red patches, and control the storage period. However, there are few reports on chemical or biological methods to control *A. arundinis*. The prolonged use of chemical fungicides will lead to the resistance of pathogenic fungi, which could potentially harm human health and environmental safety. In this regard, biocontrol agents have emerged as a potential alternative to chemical fungicides to prevent and control various diseases due to their green and pollution-free characteristics (Niem et al., 2020; Huang et al., 2021; Yuan et al., 2022). The antagonistic microorganisms that can inhibit the postharvest diseases of fruits and vegetables include species of *Candida*, *Bacillus*, *Pseudomonas*, *yeast*, and *Wickerhamomyces* (Spadaro and Droby, 2016; Kang et al., 2019; Khalifa et al., 2022). Among them, *Bacillus* has been widely isolated and utilized as an effective biocontrol agent. For example, *B. subtilis* JK14 isolated from the surface of peach (*Prunus persica* [L.] Batsch) fruits has excellent effects at controlling postharvest rot of peaches and can be used as an environmentally safe biological control agent for this purpose (Zhang et al., 2019). Similarly, the endophytic strain CC09 of *B. velezensis* is effective at controlling wheat (*Triticum aestivum* [L.]) diseases (Kang et al., 2018).

In this study, 10 strains of bacteria with antagonistic ability against *A. arundinis* were isolated from the soil of healthy sugarcane roots. Among them, one strain of *B. velezensis* designated T9 was highly effective at inhibiting *A. arundinis*, the causal agent of sugarcane mold. Overall, this research provides a novel microbial material to control sugarcane mold during postharvest storage and a theoretical basis to develop and broaden the application of microbial preservatives.

## Materials and methods

### Fungal preparation

*A. arundinis* LX1918, isolated from moldy sugarcane in our preliminary study and stored in the laboratory, was used as the research object (Liao et al., 2022). *A. arundinis* LX1918 was cultured on potato dextrose agar (PDA) at 28°C.

### Isolation of antagonistic bacterial strains

The soil was collected from the rhizosphere soil of healthy sugarcane at the Guangxi Academy of Agricultural Sciences (Nanning, China), and the bacteria in the soil samples examined were isolated by dilution plate separation (Filippi et al., 2011). A total of 10.0 g of soil was weighed and placed in a triangular bottle that contained glass beads and 90 mL of sterile water, shaken at 28°C and 180 rpm for 30 min and diluted with gradients of 10^−3^, 10^−4^, and 10^−5^. The nutrient broth (NB) plate that contained each diluent was cultured in a constant temperature incubator at 28°C for 48 h, and single colonies with obvious morphological differences were selected and purified. The purified strains were stored at 4°C for later use.

### *In vitro* screening of potential fungal biocontrol agents

The antagonistic ability of the isolated strains toward plant fungal pathogens was determined through a modified dual-culture test using *A. arundinis* LX1918 as an indicator fungus (Yu et al., 2011; Liu et al., 2021). The isolated strains were cultured overnight in LB at 180 rpm, 28°C. Under aseptic conditions, the mycelial plugs (5 mm in diameter) of a 5 days-old culture of *A. arundinis* were inoculated in the center of PDA plates, and 2 μL of a suspension of strain T9 (OD_600_ = 1.0) were inoculated 2.5 cm from the center on each side of the PDA media in petri dishes. The negative control was inoculated with the pathogenic fungal isolate. The plates were incubated at 28°C for 6 d, and the colony diameter of the pathogenic fungus of each plate was measured. The experiment was repeated three times, and each sample (all the isolates) was treated in triplicate.


$$\begin{array}{l} \mathrm{Inhibition rate}=\left(\begin{array}{l} \mathrm{colony diameter of control}-\\ \mathrm{colony diameter of treated} \end{array}\right)/\\ \left(\begin{array}{l} \mathrm{colony diameter of}\;\\ \mathrm{control}-\mathrm{cake}\;\\ \mathrm{diameter} \end{array}\right)\times 100\%\text{.} \end{array}$$

The morphology and ultrastructure of the *A. arundinis* mycelia exposed to T9 were observed by scanning electron microscopy (SEM) and transmission electron microscopy (TEM). The samples were fixed with 2.5% glutaraldehyde at 4°C for 24 h and dehydrated in an ethanol series (30, 50, 70, 80, 95, and 100%) for 20 min at each stage. After being frozen and coated with gold, the hyphae were observed under SEM (S-3400 N; Hitachi, Tokyo, Japan) and TEM (HT7700; Hitachi).

### Identification of the antagonistic bacterium T9

Strain T9 that exhibited the strongest antifungal activity was identified by morphological and molecular identification as described in Berger’s Manual of Bacterial Identification (Holt et al., 1994) and the Common Bacterial Systems Identification Manual (Dong and Cai, 2001; Vos et al., 2011). The physiological and biochemical experiments were performed on strain T9, including gram staining, growth temperature, pH tolerance, salt tolerance, starch hydrolysis, VP test, carbon source utilization, and contact enzymes among others. The experiment was performed in triplicate.

The genomic DNA was extracted and purified using a Bacterial Genome DNA Extraction Kit. The 16S rDNA, *gyrA*, and *ropB* gene sequences of strain T9 were amplified using the primers listed in Table 1. After 1% agarose gel electrophoresis, the PCR amplification products were sent for sequencing, and the sequencing results were submitted to GenBank. The comparison of similarity was performed with the known sequences in the GenBank database, and the phylogenetic tree was constructed by neighbor-joining using MEGA 7.0 (Kumar et al., 2016). The bootstrap value was set to 1,000.

**Table 1:** Primers used in this study.

Gene	Primer	Primer sequence (5′-3′)	Product/bp
16S rDNA	27F	AGAGTTTGATCCTGGCTCAG	~1,500
	1492R	TACGGCTACCTTGTTACGACTT	
gyrA	gyrA_F	CAGTCAGGAAATGCGTACGTCCTT	~1,000
	gyrA_R	CAAGGTAATGCTCCAGGCATTGCT	
ropB	rpoB_2292f	GACGTGGGATGGCTACAACT	~1,063
	rpoB_3354r	ATTGTCGCCTTTAACGATGG	
ituC	ITUC_F	CCCCCTCGGTCAAGTGAATA	594
	ITUC_R	TTGGTTAAGCCCTGATGCTC	
ItuA	ITUA_F	ATGTATACCAGTCAATTCC	1,149
	ItuA-R	GATCCGAAGCTGACAATAG	
fenB	fenB-F	CTATAGTTTGTTGACGGCTC	1,600
	fenB-R	CAGCACTGGTTCTTGTCGCA	
bmyC	bmyC-F	GAAGGACACGGCAGAGAGGTC	875
	bmyC-R	CACTGATGACTGTTCCTGCT	
bmyA	bmyA-F	AAAGCGGCTCAAGAAGCGAAACCC	1,200
	bmyA-R	CGATTCAGCTCATCGACCAGGTAGGC	
srfAA	srfAA-F	TCGGGACAGGAAGACATCAT	201
	srfAA-R	CCACTCAAACGGATAATCCTGA	
srfAB	srfAB-F	GTTCTCGCAGTCCAGCAGAAG	308
	srfAB-R	CCGAGCGTATCCGTACCGAG	

### Effect of the supernatant of *Bacillus velezensis* T9 on the mycelial growth of *Apiospora arundinis*

A suspension of strain T9 (OD_600_ = 1.0) was inoculated in a triangular flask that contained 100 mL of NB and a volume of 1% inoculum and cultured at 28°C for 72 h. The samples were then centrifuged at 3500 rpm for 20 min, and the fermentation supernatant was filtered through a 0.22 μm microporous membrane to obtain the cell-free supernatant.

PDA that contained 1, 3, 5, and 10% of the T9 cell-free supernatant was prepared at 40°C and poured into a plate. Each plate was inoculated with *A. arundinis* plugs (5 mm in diameter). The culture was repeated three times at 28°C for 6 d with three replicates. The colony diameter was measured by cross-over trials, and the mycelial growth rate and rate of inhibition of each treatment were calculated using the following equations:


$$\begin{array}{l} \mathrm{Mycelium growth rate}=\left(\begin{array}{l} \mathrm{treated colony diameter}-\\ \mathrm{cake diameter} \end{array}\right)/\\ \mathrm{culture days}\text{.} \end{array}$$

$$\begin{array}{l} \mathrm{Mycelium}\;\\ \mathrm{growth}\;\\ \mathrm{inhibition rate}=\left(\begin{array}{l} \mathrm{control mycelium growth rate}-\\ \mathrm{treatment mycelium growth rate} \end{array}\right)/\\ \left(\begin{array}{l} \mathrm{control mycelium}\;\\ \mathrm{growth rate} \end{array}\right)\times 100\%\text{.} \end{array}$$

Approximately 100 mL of potato dextrose broth (PDB) that contained 1, 3, 5, and 10% T9 cell-free supernatant was prepared, and 100 μL of an *A. arundinis* spore suspension (1 × 10^5^ spores mL^−1^) was added to the PDB and cultured at 28°C, 180 rpm for 7 d. Cultures that lacked T9 cell-free supernatant were used as the control. The treatment was performed in triplicate. The PDB was filtered through four layers of sterile gauze to obtain the wet hyphae. The hyphae were dried at 50°C and weighed. The amount of mycelial growth and its rate of inhibition were calculated using the following equations:


$$\begin{array}{l} \mathrm{Amount of mycelium growth}=\mathrm{dry}\;\mathrm{weight of the treated}\;\\ \mathrm{mycelium}/\mathrm{culture days}\text{.} \end{array}$$

$$\begin{array}{l} \mathrm{Mycelium}\;\\ \mathrm{growth}\;\\ \mathrm{inhibition rate}=\left(\begin{array}{l} \mathrm{control mycelium growth}-\\ \mathrm{treatment mycelium growth} \end{array}\right)/\\ \mathrm{control mycelium}\;\\ \mathrm{growth}\times 100\%\text{.} \end{array}$$

### Control effect of *Bacillus velezensis* strain T9 on sugarcane infected with *Apiospora arundinis*

The ability of strain T9 to control disease on sugarcane infected with *A. arundinis* was identified using sugarcane stems as previously described with minor modifications (Huang et al., 2021). Wounds (3 mm deep and 5 mm wide) were made on each sugarcane stem using a sterile borer. Each wound was injected with a 1 × 10^8^ CFU/mL suspension of *B. velezensis* T9 (100 μL) or supernatant, while sterile water and protamine were used in the negative and positive control group, respectively. After standing for 24 h, the *A. arundinis* cake with a diameter of 5 mm was inoculated on the sugarcane wound, and the diameter of lesions was measured with Vernier calipers on days 5 and 10. A small piece of the sugarcane tissue (3 mm × 3 mm) was taken from the distal end of the disease site, disinfected with 75% ethanol, and washed with sterile water. Finally, the tissue was placed on PDA and cultured in a 28°C incubator for 5 d.

### Inhibitory effect of *Bacillus velezensis* T9 on the production of 3-NPA By *Apiospora arundinis*

The suspension of *A. arundinis* spores (10^5^ CFU/mL) was added to PDB that contained 1, 3, 5, or 10% of cell-free supernatant from strain T9, and the cultures were incubated at 28°C for 15 d with shaking at 140 rpm. A volume of 2 mL of the PDB culture filtered through four layers of gauze was added with 10 mL of acetonitrile and then vortexed for 10 min. After a period of time, 1.5 g of NaCl was added to the mixture after ultrasonic extraction for 20 min, vortexed for 2 min, and centrifuged at 4000 rpm for 5 min. Afterward, 5 mL of the cell-free supernatant was taken and evaporated under a stream of N_2_, and the residue was dissolved in 1 mL of distilled water. It was then passed through a filtration membrane. The filtrate was analyzed using HPLC (Waters, Milford, MA, United States) coupled to a two-stage array detector and an Agilent XDB-C18 column (250 × 4.6 mm, 5 μm particle size) (Agilent Technologies, Santa Clara, CA, United States). The mobile phase (phosphoric acid: methanol [97: 3]) was pumped at a flow rate of 1.0 mL/min. The detection wavelength was 208 nm. The amount of 3-NPA produced by the three fungal isolates was calculated by determining the peak area of the compound in the chromatogram and comparing it to a known standard (Wei et al., 1994). The rate of inhibition of 3-NPA was calculated using the following equation:


$$\begin{array}{l} \mathrm{Rate of}\;3-NPA\;\mathrm{inhibition}=\left(\begin{array}{l} 3-NPA\;\mathrm{production of the control}-\\ 3-NPA\;\mathrm{production of}\;\\ \mathrm{the suspension treatment} \end{array}\right)/\\ 3-NPA\;\mathrm{production of the control}\times 100\%\text{.} \end{array}$$

### Effect of VOCs on the mycelial growth of *Apiospora arundinis*

The inhibitory effects of the volatile organic compounds (VOCs) produced by strain T9 on the growth of *A. arundinis* were studied using the two-sealed-base-plates method (Gao et al., 2017; Grzegorczyk et al., 2017).

A bacterial T9 suspension (10^8^ cells/mL) was prepared, and 300 μL of the suspension was seeded on a nutrient agar (NA) plate. After 2 h, a 5 mm-diameter plug of *A. arundinis* was placed on the center of the PDA plate. The two plates were covered and sealed with polyethylene stretch wrap and then cultured at 28°C for 6 d. The NA plate without T9 inoculation was used as the control, and each treatment was performed in triplicate. The colony diameter of the pathogenic fungus was measured (cross-crossing method), and the rate of inhibition was calculated as follows:


$$\begin{array}{l} \mathrm{Rate of}\;\\ \mathrm{inhibition}=\left(\begin{array}{l} \mathrm{colony diameter of the}\;\\ \mathrm{control group}-\mathrm{colony}\;\\ \mathrm{diameter of the treated group} \end{array}\right)/\\ \left(\begin{array}{l} \mathrm{colony diameter of the}\\ \;\mathrm{control}-\mathrm{the cake}\;\\ \mathrm{diameter} \end{array}\right)\times 100\%\text{.} \end{array}$$

### Biofilm formation by the antagonists

The formation of solid surface-associated biofilm was quantified using the crystal violet staining method with minor modifications (Parafati et al., 2015; Zhang et al., 2020). A suspension of strain T9 (10^8^ CFU/mL) was inoculated into glass tubes that contained 5 mL of LB. Then, the tubes were incubated at 28°C for 3 h, 24 h, 48 h, and 72 h in a shaker at 75 rpm. The cultures under the biofilm in the tubes were carefully removed, leaving only the biofilm, and dried at room temperature. Later, 3.0 mL of 0.1% (w/v) crystal violet was added to each tube for 30 min. The crystal violet was suctioned out, rinsed 10 times with distilled water, and decolorized with 3.0 mL of 95% ethanol. Finally, the solution was measured at 570 nm. Each treatment was performed in triplicate.

### Colonization of *Bacillus velezensis* T9 in sugarcane wounds

Artificial wounds were made on superficially sanitized sugarcane stems using a hole punch, and 80 μL of a suspension of T9 cells (10^8^ CFU/mL) was inoculated into the wound. The sugarcane stems were then incubated at 25°C. Later, the wounded tissue samples were collected using a sterile knife at various times (3 h and 1, 2, 3, 4, 5, 6, 7, 8, 9, and 10 d) after inoculation and weighed. The wounded tissues were then ground. Serial dilutions of each sample were prepared, and the dilutions were plated onto NA. Finally, the bacterial colonies were counted after 2 d of incubation at 28°C, and three replicates were used for each treatment. The experiment was repeated twice.

### Detection of enzyme activity and the secondary metabolites of strain T9

A 1 μL suspension of strain T9 (OD_600_ = 1) was spotted onto agar plates that contained skim milk, carboxymethyl cellulase, and β-1,3-glucanase and cultured at 28°C for 2–4 d to determine the ability of strain T9 to produce and secrete cell wall lytic enzymes, including cellulase, glucanase, and protease (Shakeel et al., 2015). The ability of antagonists to produce various hydrolases was evaluated by observing the size of the hydrolase circle around the colony. The hydrolysis circle of cellulase was observed after staining with Congo red (Nagpure et al., 2014; Zhou et al., 2022). The ability of *B. velezensis* T9 to produce siderophores was determined by observing chrome azurol S (CAS) medium. A suspension of 1 μL of T9 (OD_600_ = 1) was inoculated onto CAS plates and cultured at 28°C. A yellow-orange zone that formed after 10 d was considered to indicate the presence of a siderophore.

### Statistical analysis

All the assays were performed in triplicate, and the results are presented as the mean ± SD. The significant differences between the mean values were determined by a one-way analysis of variance (ANOVA) using Duncan’s multiple range test (*p* < 0.05). The statistical analyses were performed with SPSS.

## Results

### Isolation of the antagonistic bacteria

**Figure 1:**
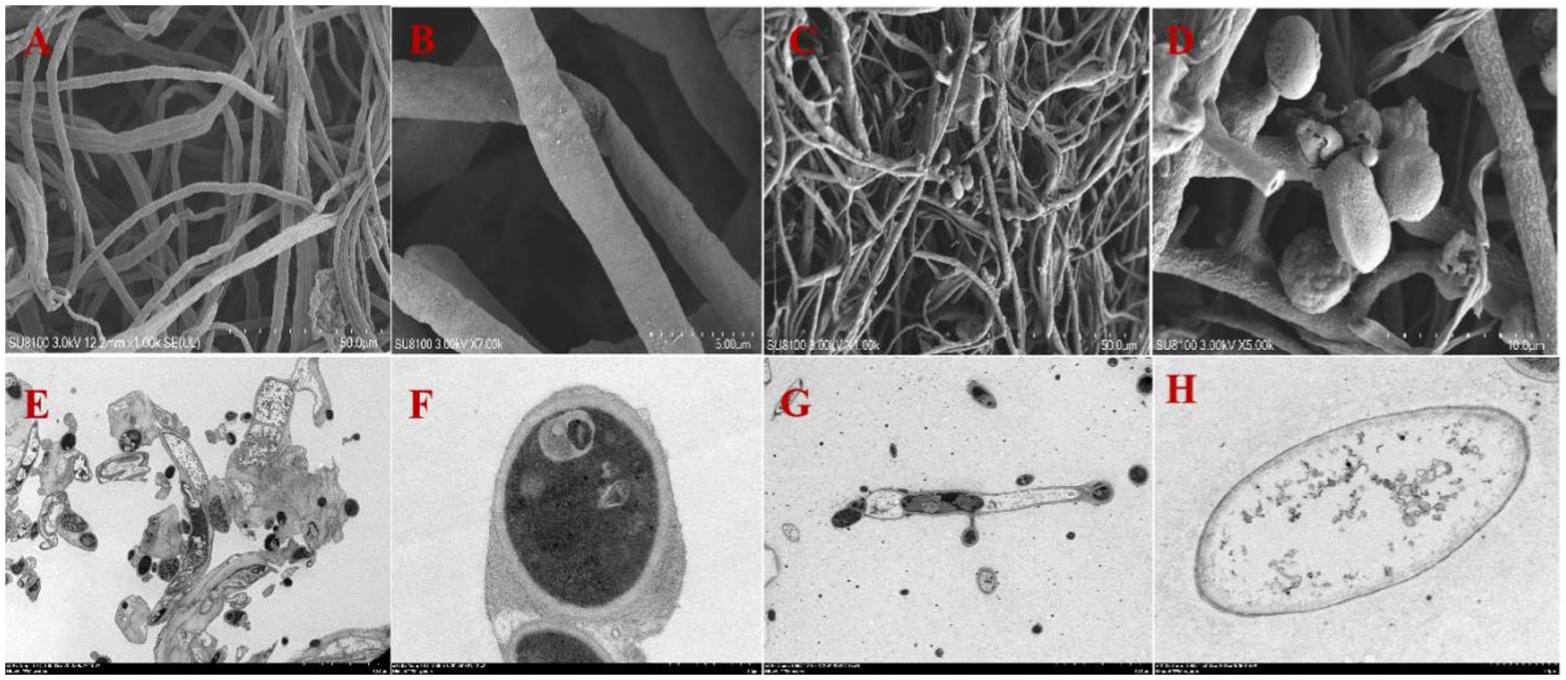
Mycelial morphology and ultrastructure observation of *A. arundinis* using SEM and TEM. SEM of the control group hyphae **(A,B)**, and hyphae treated with strain T9 group **(C,D)**. TEM of the control group **(E,F)**, and treated with strain T9 group **(G,H)**. The micrographs were taken at the magnification of **(A,C)** 1,000×, **(B)** 7,000×, **(D)** 5,000×, **(E,G)** 1,500× **(F,H)** 15,000×, respectively.

A total of 10 strains with antagonistic effects on *A. arundinis* were isolated from the soil. Among them, T9 showed an average rate of inhibition of 78.8% with a zone of inhibition of 9.1 mm in diameter (Supplementary Figure S1). The antagonistic effect of strain T9 on *A. arundinis* was assessed by scanning electron microscopy (SEM) (Figure 1), which showed that the mycelia were round with uniform thickness and a smooth surface in the control group. In contrast, the mycelia treated with T9 became thinner, shriveled, and folded, and the surface integrity of the mycelia was damaged. The spores were also shriveled and deformed. Transmission electron microscopy (TEM) showed that the untreated mycelia were full, and the fungal wall was complete, with a uniform thickness and dense structure. Additionally, the fungal membrane was intact, and no cytoplasmic wall separation was observed. In contrast, the treated mycelia were deformed and disintegrated. The cell wall showed a fuzzy structure with uneven thickness, and the surface mucous layer was thin and discrete outside of the cell wall. The cell membrane was blurred and thin with perforations, and there was severe separation of the cytoplasm from the cell wall. Most of the organelles were disintegrated and appeared as irregular fragments. The vacuole was contracted, and the thallus was poor with signs of breakage and disintegration.

### Identification of the antagonistic bacterium T9

The growth of strain T9 cultured in the NA medium is shown in Supplementary Figure S2. The morphological observation showed that the single colonies of T9 were milky white, dried, not smooth, and with a wrinkled surface that developed as the culture time increased. The gram stain was positive, and the bacteria were rod-shaped with slightly rounded ends. The physiological and biochemical assays showed that T9 could utilize sucrose, glucose, D-sorbitol, D-fructose, D-mannitol, proline, and alanine. Contact enzyme reaction, gelatin liquefaction, VP test, methyl red test, and starch hydrolysis were positive reactions, while citrate utilization was a negative reaction (Supplementary Table S1). These characteristics were consistent with those of the proximate *Bacillus* spp.

The partial 16S rDNA, *gyrA*, and *rpoB* gene sequences from strain T9 were amplified and submitted to the GenBank database with the gene entry numbers OR241131, OR326857, and OR296710, respectively, to verify the classification status of T9. A BLAST analysis of strain T9 showed that these three sequences had more than 99% homology with *B. velezensis* in GenBank. Phylogenetic trees based on 16S rDNA, *gyrA*, and *rpoB* gene sequences were constructed using MEGA 7.0, and the results showed that the 16S rDNA, *gyrA*, and *rpoB* sequences of strain T9 all clustered into a lineage with *B. velezensis* (Supplementary Figure S3). Based on the morphological, physiological, and biochemical characteristics and sequence analysis, strain T9 was identified as *B. velezensis*.

### Effect of the T9 supernatant on the mycelial growth of *Apiospora arundinis*

**Figure 2:**
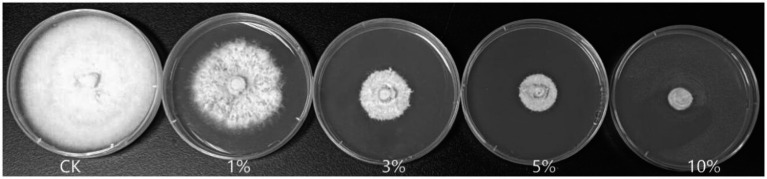
Inhibition test of *B. velezensis* T9 cell-free supernatant on *A. arundinis*. Strain T9 suspension was centrifuged at 3500 rpm for 20 min, the supernatant was filtered through a 0.22 μm microporous membrane to obtain the cell-free supernatant, PDA medium that contained 1, 3, 5, and 10% of the T9 cell-free supernatant were inoculated with *A. arundinis* plugs, with untreated PDA as the control.

As shown in Figure 2 and Table 2, the 3, 5, and 10% fermentation filtrate significantly inhibited the growth of *A. arundinis*, with rates of inhibition of 66.54, 73.78, and 82.66%, respectively, on the PDA plates that contained T9 fermentation solution. The rates of inhibition of 1, 3, 5, and 10% fermentation filtrate for the dry weight of *A. arundinis* were 6.64, 30.62, 76.38, and 99.63%, respectively. The fermentation filtrate of *B. velezensis* T9 had a significant inhibitory effect on the diameter and amount of growth of the mycelia, but the inhibitory effect of the fermentation filtrate on the amount of growth of *A. arundinis* was slightly better than that of the mycelial growth rate.

**Table 2:** Inhibition of cell-free fermentation of *B. velenzensis* T9 on hyphal growth of *A. arundinis*.

Treatment	Hyphal diameter		Hyphal dry weigh	
	Growth speed (mm/d)	Inhibition rate (%)	Increase speed (mg/d)	Inhibition rate (%)
1% fermentation filtrate	8.04 ± 0.075a	39.10	140.56 ± 4.19a	6.64
3% fermentation filtrate	4.42 ± 0.22b	66.54	104.44 ± 10.84b	30.63
5% fermentation filtrate	3.46 ± 0.37c	73.78	35.56 ± 3.85c	76.38
10% fermentation filtrate	2.29 ± 0.11d	82.66	0.56 ± 0.96d	99.63
Control	13.20 ± 0.017a	–	150.56 ± 6.74a	–

Data are expressed as means of three replicates ± SD. Values in the two columns followed by different letters are significantly different (*p* < 0.05).

### Effect of the T9 supernatant on the production of 3-NPA by *Apiospora arundinis*

Table 3 shows the effects of 1, 3, 5%, or 10% of the cell-free supernatant of strain T9 on the production of 3-NPA. With the increase in the amount of supernatant, the effect on the production of 3-NPA became more significant. Compared to the control, 1% of the strain T9 supernatant had little effect on the production of 3-NPA (9.37 μg/mL), and the rate of inhibition by 3-NPA was 21.81%. The production of 3-NPA could not be detected following treatment with 5 and 10% of the supernatant of strain T9.

**Table 3:** 3-NPA production and 3-NPA inhibition ratio as affected by cell-free supernatant of *B. velenzensis* T9.

Treatments	3-NPA (ug/mL)	Inhibition rate (%)
The control	12.92	–
10% fermentation filtrate	0	100
5% fermentation filtrate	0	100
3% fermentation filtrate	3.12	75.85
1% fermentation filtrate	7.37	42.96

### Inhibitory effects of the VOCs on *Apiospora arundinis*

The average colony diameter of *A. arundinis* exposed to the VOCs produced by *B. velezensis* T9 was 29.0 mm after 6 d, which was 70.0% smaller than that of the unexposed samples. In addition, the mycelia that were not exposed to the VOCs grew dense, while those of the exposed sample grew extremely poorly. The VOCs produced by *B. velezensis* T9 significantly inhibited the growth of *A. arundinis* mycelia *in vitro*.

### Determination of the formation of biofilm by strain T9

**Figure 3:**
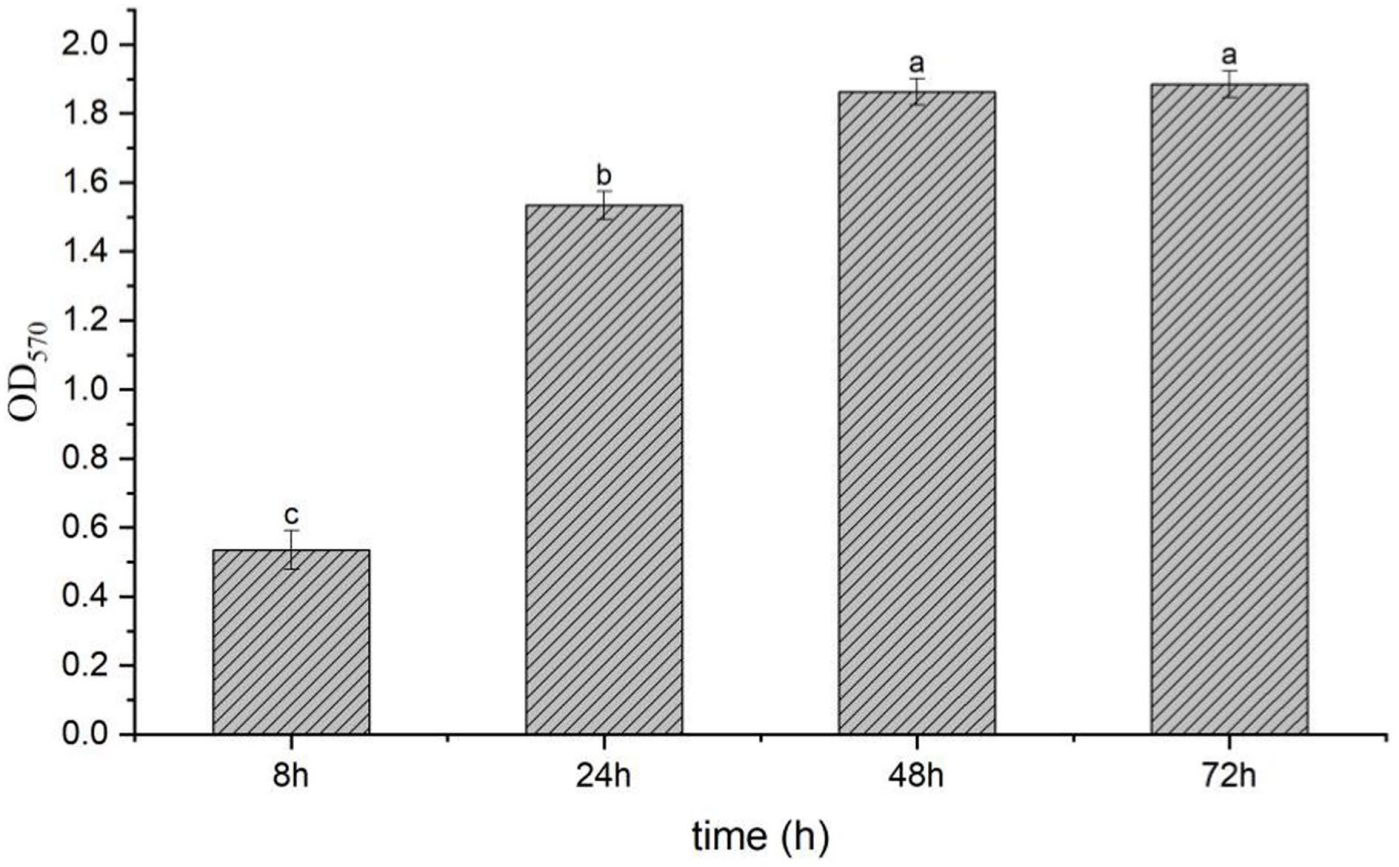
Biofilm formation of *B. velezensis* T9 using the crystal violet staining method. The absorption value of OD_570_ were used to represent the yield of biofilm formation by *B. velezensis* T9. Data were expressed as the mean of three replicates ±SD. Significant differences (*p* < 0.05) between means are indicated by letters above the histogram bars.

The result showed that the LB without strain T9 was clear and transparent; the surface was clean, and no biofilm formed after standing for 72 h. After the incubation of the bacterial solution for 8 h, a thin layer of biofilm formed on the surface of the bacterial solution with an OD_570_ value of 0.53. After incubation of the bacterial solution for 24 h, a significantly thicker bacterial film was formed on the surface, with an OD_570_ value of 1.53. This was significantly higher than the amount that formed at 8 h. The bacterial film reached its peak at 48 h, with an OD_570_ value of 1.86. The thick bacterial film was still maintained at 72 h, with no significant difference from 48 h (Figure 3). Thus, *B. velezensis* has a strong ability to form biofilms, which could not be degraded in a short time.

### Colonization of *Bacillus velezensis* T9 in the sugarcane wounds

**Figure 4:**
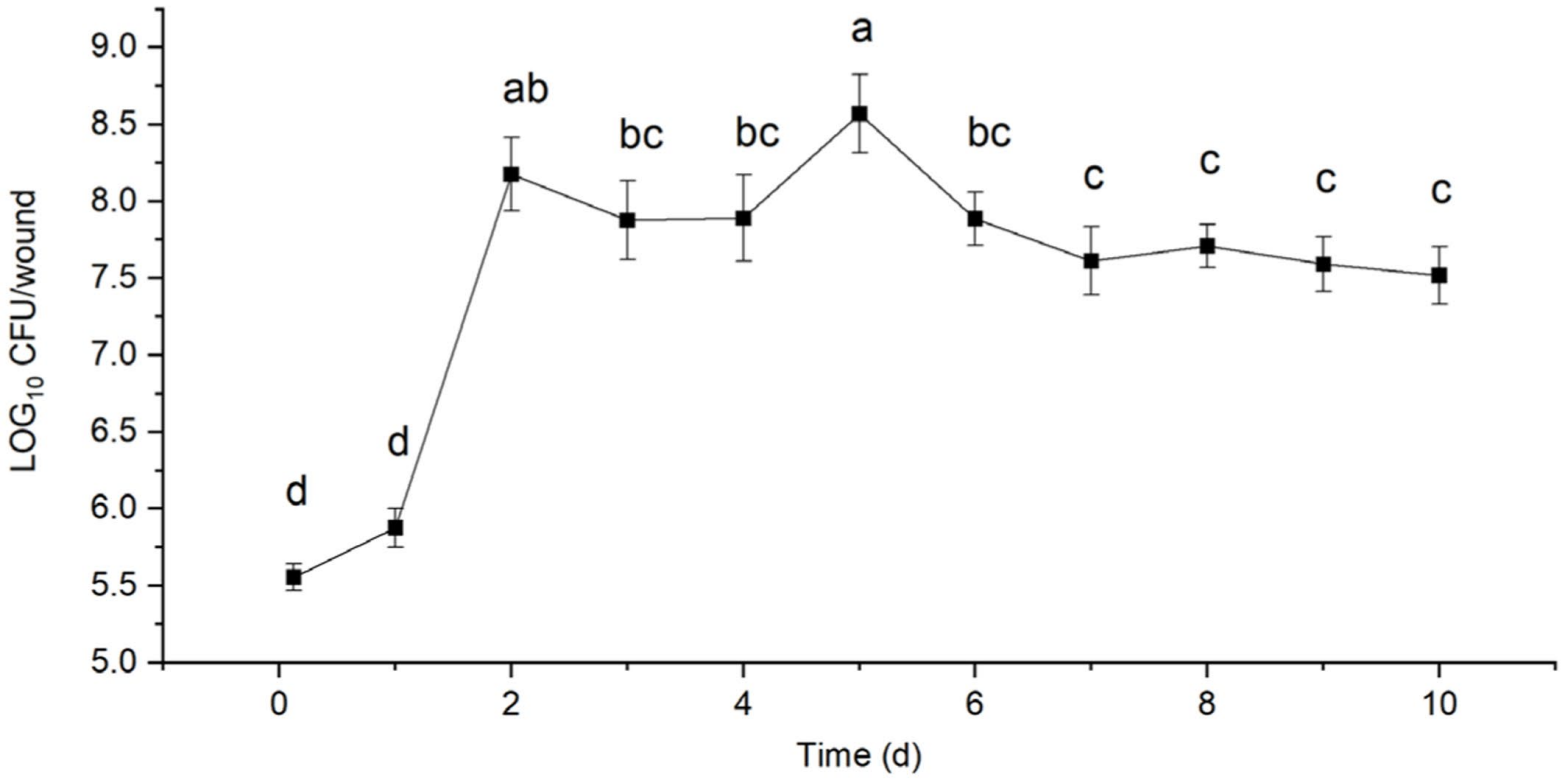
Population dynamics of *B. velezensis* strain T9 in wounds tissue of sugarcane stems at 25°C for 10 d. Data were expressed as the mean of three replicates ±SD. Significant differences (*p* < 0.05) between means are indicated by letters above the histogram bars.

After inoculation with *B. velezensis* strain T9, the treated sugarcane stems were cultured in an incubator at 25°C and 90% relative humidity. A total of 3.65 × 10^5^ CFU of strain T9 colonized the sugarcane stems after 3 h (0 d) of inoculation of the sugarcane stems, which rapidly increased to 7.75 × 10^5^ CFU after 1 d. The population of strain T9 colonies reached a small peak with 1.63 × 10^8^ CFU after 2 d. They declined slightly but remained stable after 3 and 4 d of treatment. The total number of colonies reached their highest peak at 4.17 × 10^8^ CFU after 5 d. Subsequently, the number of colonies decreased, and after 10 d of treatment, the number of colonies tended to be stable and maintained 3.50 × 10^7^ CFU, which was still higher than the initial number (Figure 4).

### Detection of enzyme activity and the secondary metabolites of strain T9

**Figure 5:**
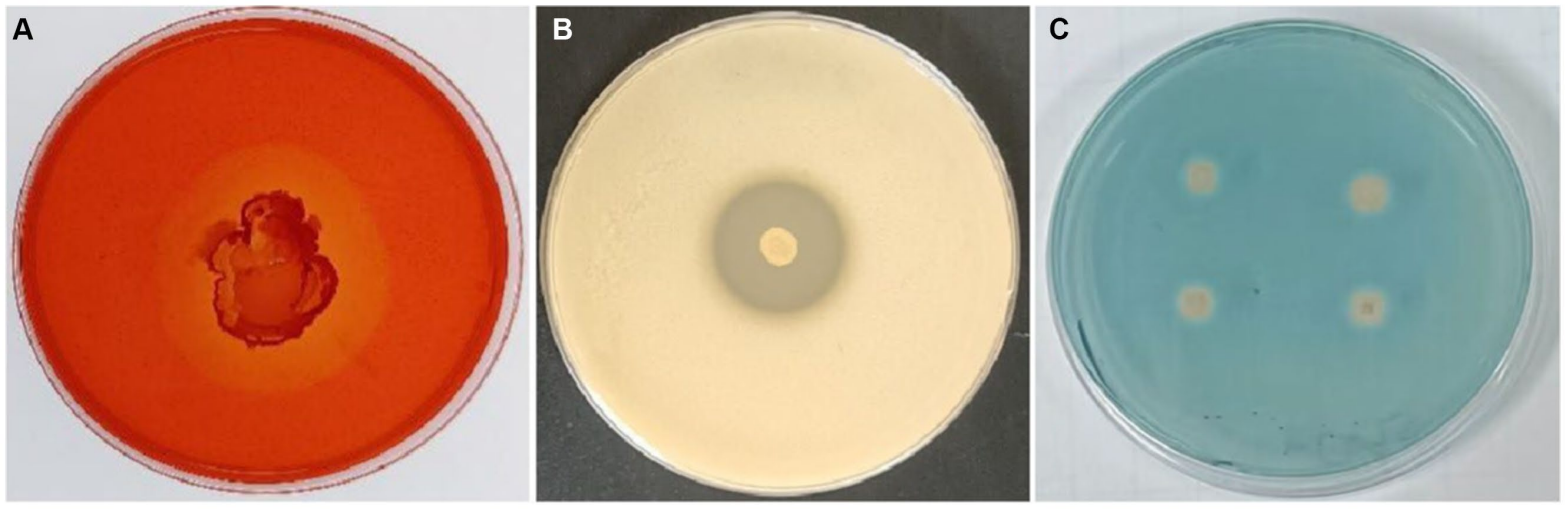
Detection of secretase activity and secondary metabolites in *B. velezensis* strain T9. **(A)** Cellulase detection, **(B)** protease detection, and **(C)** siderophore detection.

The ability of *B. velezensis* T9 to produce cell wall lytic enzymes and secondary metabolites was studied to characterize its mechanism of antagonism. This strain formed translucent hydrolysis circles in skim milk and carboxymethyl cellulose (CMC) media, indicating that *B. velezensis* T9 could produce protease and cellulase, respectively. Additionally, *B. velezensis* T9 changed the CAS medium from blue to light orange, indicating that T9 was capable of producing siderophores (Figure 5).

### Effect of *Bacillus velezensis* strain T9 against *Apiospora arundinis* in sugarcane

**Figure 6:**
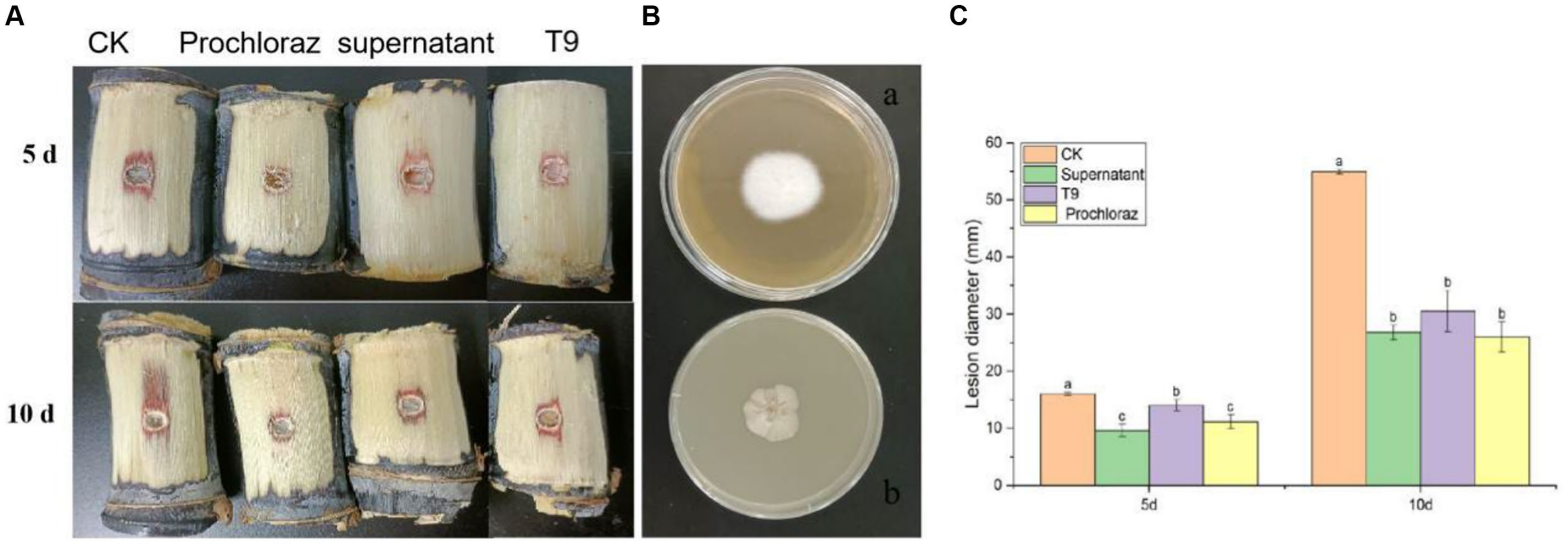
Effect of strain T9 cell suspension and supernatant on sugarcane Apiospora mold disease lesion diameter. **(A)** Strain T9 cell suspension and supernatant antagonized sugarcane Apiospora mold. Treatment with sterile distilled water or protamine was used as the negative and positive control. **(B)** The morphological observation of control group **(A)** and T9 treatment **(B)**. **(C)** Statistical analysis of lesion diameter. Disease lesion length were measured at 5 and 10 day, respectively. Data were expressed as the mean of three replicates ±SD. Significant differences (*p* < 0.05) between means are indicated by letters above the histogram bars.

The sugarcane stems were treated with a suspension of strain T9 cells or its cell-free supernatant and inoculated with *A. arundinis* to study the efficacy of strain T9 in controlling Apiospora mold. The wounds were observed on days 5 and 10 (Figure 6A). Compared with the control, the diameters of 5 days-old lesions of sugarcane treated with the T9 cell suspension or its cell-free supernatant decreased by 18.9 and 39.9%, respectively, while the 10 days-old lesion decreased by 45.4 and 51.2%, respectively. The average lesion diameter on the sugarcane stems treated with strain T9 cell suspension or its cell-free supernatant decreased significantly but had no significant difference than treatment with the fungicide prochloraz (Figure 6C). Additionally, as shown in Figure 6B, *A. arundinis* was not re-isolated from the sugarcane stems treated with T9, culture filtrates, and prochloraz but was re-isolated from the control group. These findings indicated that T9 treatment and its culture filtrates significantly inhibited the effects on Apiospora mold caused by *A. arundinis*.

## Discussion

The 3-NPA toxin produced by moldy sugarcane spoiled with *Apiospora* poses a potential threat to human health. Therefore, developing biological agents is critical for biological control. In recent years, biological control agents (BCAs) have attracted increasing attention due to their green and safe characteristics (Wang et al., 2016; Hu et al., 2021). Among them, *Bacillus* is the most widely used type of biocontrol bacteria, with strong stress resistance, good biosafety, and other outstanding advantages (Lastochkina et al., 2019; Rivas-Garcia et al., 2019; Zhou et al., 2019). However, to our knowledge, there is no report on the use of *B. velezensis* for the biological control of Apiospora mold on sugarcane caused by *A. arundinis*. In this study, *B. velezensis* strain T9 was isolated from the rhizosphere soil of healthy sugarcane and displayed potent biocontrol activity that enabled it to effectively control Apiospora mold caused by *A. arundinis* on sugarcane. Relevant studies showed that *B. velezensis* has been considered as a biocontrol agent to inhibite the growth of the major fungal plant pathogens. Myo et al. reported that *B. velezensis* NKG-2 could be used for bio-control activities against fungal diseases and potential plant growth promotion (Myo et al., 2019).

The cell suspension of strain *B. velezensis* T9 and its cell-free supernatant significantly inhibited the growth of *A. arundinis* mycelia *in vitro*. SEM and TEM showed that *B. velezensis* T9 had significant inhibitory effects on the mycelia and cells of *A. arundinis*, which led to disordered growth, atrophy, wrinkles, and distortion of the hyphae and changes in spore morphology, while damaging the cell membrane and inducing protoplasm leakage and eventual cell death. The diameters of the lesions on sugarcane stems treated with *B. velezensis* T9 cell suspensions and the supernatant of cell-free cultures were significantly lower than those of the control group, which inhibited the growth of *A. arundinis* and 3-NPA production. This result was consistent with those of previous research. For example, *B. subtilis* ABS-S14 and its cell-free supernatant had strong antagonistic effects on the hyphal growth of *Penicillium digitatum*, the causal agent of green mold on citrus (Waewthongrak et al., 2015). *B. velezensis* strain P2-1 isolated from apple (*Malus domestica* Borkh.) branches and its cell-free supernatant exhibited strong inhibitory effects on the growth of *Botryosphaeria dothidea*, which resulted in hyphal deformity (Yuan et al., 2022). Additionally, the biocontrol mechanism of *B. velezensis* T9 against *A. arundinis* was studied.

The rapid growth and high population density of antagonists in the wounded tissues could the plants or fruits from pathogens by competing with them for space and nutrients, thus, preventing the ingress of pathogenic microorganisms (Zhang et al., 2008; Liu et al., 2013; Zeriouh et al., 2014). Nunes et al. (2008) reported that the decay of oranges (*Citrus sinensis* [L.] Osbeck) treated with the bacterium *Pantoea agglomerans* before refrigeration, i.e., stored at 20°C for 24 h, was more effective with more than a 10-fold increase in *P. agglomerans* compared to fruits that were immediately refrigerated. In this study, the population of strain T9 colonies reached a peak after 3 d, which was greater than the population at 0 d after 10 d of treatment but retained its ability to colonize at a high density and had a good potential to act as a BCA. Further control experiments showed that the use of strain T9 significantly reduced the degree of damage by sugarcane mold compared with the control group, and its effect at controlling this disease was consistent with that of the imidamide class of chemical pesticides. Thus, this strain has strong potential for application in the field and lays a foundation for the development of BCAs.

In recent years, bacterial VOCs have been shown to play an imperative role in microbial interactions, and bacterial-plant interactions have received much attention. Many studies have reported that *B. velezensis* can produce VOCs that have strong inhibitory activity on the growth of bacteria and plant pathogens, and the VOCs produced by *B. velezensis* ZSY-1 significantly inhibited six types of plant pathogenic fungi (Gao et al., 2017). The VOCs produced by *B. velezensis* inhibited the growth of *C. gloeosporioides* and controlled anthracnose on mango (*Mangifera indica* Linn) (Yang et al., 2023). The VOCs produced by *B. velezensis* T9 could also serve as potential biocontrol agents against fungal diseases.

Biofilms are essential community structures of bacteria and play an important role in the mechanism of antagonism against plant pathogenic fungi (Bogino et al., 2013). The formation of *Bacillus* biofilm primarily includes adhesion development, maturity, and dispersion dissociation. In this study, T9 formed a thin layer of biofilm at 8 h. The amount of biofilm formed at 24 h increased significantly compared with that 8 h, and the amount of biofilm formed at 72 h increased slightly compared with the 48 h treatment. However, the difference was not significant. This period is the stable period of biofilm formation, which indicated that T9 is effective at forming biofilm and can promote its colonization in sugarcane.

The secretion of extracellular hydrolases is one of the most important biocontrol mechanisms of antagonistic strains of *Bacillus* (Xu et al., 2016; Subbanna et al., 2018). Dextran, chitin, cellulose, and protein are the primary components of fungal cell walls. It has been shown that chitinase, glucanase, cellulase, and protease play a vital role in controlling plant diseases. In this study, antagonist T9 was found to produce cellulase and protease. Therefore, it was hypothesized that this antagonist can inhibit *A. arundinis* by acting on the cell wall by changing or degrading it and destroying the integrity of the cell. In addition to increasing the supply of iron to plants and microorganisms, siderophores could inhibit the growth of plant pathogens (Xue et al., 2013).

## Conclusion

In summary, *B. velezensis* strain T9 isolated from the soil of healthy sugarcane exhibited the strongest biocontrol activity against Apiospora mold caused by *A. arundinis* on postharvest sugarcane during storage. The results showed that the formation of biofilm, strong ability to colonize sugarcane tissue, and the production of volatile organic compounds (VOCs), cellulase, protease might be the primary mode of antagonizing Apiospora mold on postharvest sugarcane. Overall, these findings provide a novel biocontrol agent to prevent and control Apiospora mold caused by *A. arundinis* on postharvest sugarcane during transportation and storage. To the best of our knowledge, this is the first study that reports the strong antifungal activities of *B. velezensis* toward *A. arundinis*.

## Data availability statement

The datasets presented in this study can be found in online repositories. The names of the repository/repositories and accession number(s) can be found below: NCBI-OR241131, OR326857, and OR296710.

## Author contributions

JL: Writing – original draft, Writing – review & editing. XL: Writing – review & editing. HL: Writing – review & editing. LM: Writing – review & editing. RM: Writing – review & editing. WC: Writing – review & editing. YW: Writing – review & editing. TW: Writing – review & editing. WJ: Writing – review & editing.

## Supplementary material

SUPPLEMENTARY FIGURE S1 Antifungal activity of strain T9 against *A. arundinis*. **(A)** Antifungal activity of strain T9 against *A. arundinis*. **(B)** untreated *A. arundinis*.

SUPPLEMENTARY FIGURE S2 Colony morphological characteristics of the strain T9. **(A)** Strain T9 colony morphology. **(B)** Gram staining of thestrain T9.

SUPPLEMENTARY FIGURE S3 Neighbor-joining phylogenetic tree of the strain T9. Neighbor-joining phylogenetic tree of the strain T9 were constructed by MEGA7.0 basing on 16 S rDNA **(A)**, gyrA **(B)**, and ropB **(C)**. The numbers at the branches indicate the confifidence level calculated by bootstrap analysis (1000).

## References

[ref1] Birkelund T. Johansen R. F. Illum D. G. Dyrskog S. E. Østergaard J. A. (2021). Fatal 3-Nitropropionic acid poisoning after consuming coconut water. Emerg. Infect. Dis. 27, 278–280. doi: 10.3201/eid2701.202222, PMID: 33350928 PMC7774558

[ref2] Bogino P. C. De Las Mercedes Oliva M. Sorroche F. G. Giordano W. (2013). The role of bacterial biofilms and surface components in plant-bacterial associations. Int. J. Mol. Sci. 14, 15838–15859. doi: 10.3390/ijms140815838, PMID: 23903045 PMC3759889

[ref3] Dhansu P. Ram B. Singh A. K. Tomar S. K. Karuppaiyan R. Kumar R. (2023). Different treatments for sugarcane juice preservation. Foods 12:311. doi: 10.3390/foods12020311, PMID: 36673403 PMC9857402

[ref4] Dong X. Z. Cai M. Y. (2001). Manual for systematic identification of common bacteria. Beijing: Science Press, 364–398

[ref5] Filippi M. C. C. Da Silva G. B. Silva-Lobo V. L. Côrtes M. V. C. Moraes A. J. G. Prabhu A. S. (2011). Leaf blast (Magnaporthe oryzae) suppression and growth promotion by rhizobacteria on aerobic rice in Brazil. Biol. Control 58, 160–166. doi: 10.1016/j.biocontrol.2011.04.016

[ref6] Gao Z. Zhang B. Liu H. Han J. Zhang Y. (2017). Identification of endophytic bacillus velezensis zsy-1 strain and antifungal activity of its volatile compounds against alternaria solani and botrytis cinerea. Biol. Control 105, 27–39. doi: 10.1016/j.biocontrol.2016.11.007

[ref7] Grzegorczyk M. Żarowska B. Restuccia C. Cirvilleri G. (2017). Postharvest biocontrol ability of killer yeasts against Monilinia fructigena and Monilinia fructicola on stone fruit. Food Microbiol. 61, 93–101. doi: 10.1016/j.fm.2016.09.005, PMID: 27697174

[ref8] Hajnal E. J. Kos J. Malachová A. Steiner D. Stranska M. Krska R. (2020). Mycotoxins in maize harvested in Serbia in the period 2012–2015. Part 2: non-regulated mycotoxins and other fungal metabolites. Food Chem. 317:126409. doi: 10.1016/j.foodchem.2020.126409, PMID: 32087516

[ref9] He F. Zhang S. Qian F. Zhang C. (1995). Delayed dystonia with striatal CT lucencies induced by a mycotoxin (3-nitropropionic acid). Neurology 45, 2178–2183. doi: 10.1212/WNL.45.12.2178 8848189

[ref10] Holt J. G. Krieg N. R. Sneath P. H. A. Staley J. T. Williams S. T. (1994). Bergey’s manual of determinative bacteriology. Baltimore, MD: Williams and Wilkins Press, 195–256

[ref11] Hou Z. Z. Liu G. H. Song L. J. Ren X. R. Yan Y. X. (1989). Isolation and toxicity of fungi from moldy sugarcane. Chin. J. Public Health 6, 341–342.

[ref12] Hu Y. Li Y. Yang X. Li C. Wang L. Feng J. (2021). Effects of integrated biocontrol on bacterial wilt and rhizosphere bacterial Community of Tobacco. Sci Rep-Uk. 11:2653. doi: 10.1038/s41598-021-82060-3, PMID: 33514837 PMC7846572

[ref13] Hu W. J. Liang X. T. Chan H. M. Wang Y. H. Liu X. J. Luo X. Y. (1986). Isolation and identification of toxic substance-3-nitropropionic acid from degenerated sugarcane arthrobotrys sp. Chin. J. Prevent. Med. 6, 321–323.

[ref14] Huang Y. Sun C. Guan X. Lian S. Li B. Wang C. (2021). Biocontrol efficiency of Meyerozyma guilliermondii Y-1 against apple postharvest decay caused by Botryosphaeria dothidea and the possible mechanisms of action. Int. J. Food Microbiol. 338:108957. doi: 10.1016/j.ijfoodmicro.2020.108957, PMID: 33221041

[ref15] Kang X. Wang L. H. Guo Y. Arifeen M. Z. U. Liu C. (2019). A comparative transcriptomic and proteomic analysis of Hexaploid Wheat's responses to colonization by bacillus velezensis and Gaeumannomyces graminis, both separately and combined. Mol Plant Microbe 32, 1336–1347. doi: 10.1094/MPMI-03-19-0066-R, PMID: 31125282

[ref16] Kang X. Zhang W. Cai X. Zhu T. Xue Y. Liu C. (2018). Bacillus velezensis CC09: a potential vaccine for controlling wheat diseases. Mol. Plant-Microbe Interact 31, 623–632. doi: 10.1094/MPMI-09-17-0227-R, PMID: 29372814

[ref17] Khalifa M. W. Rouag N. Bouhadida M. (2022). Evaluation of the antagonistic effect of pseudomonas Rhizobacteria on Fusarium wilt of chickpea. Agriculture 12:429. doi: 10.3390/agriculture12030429

[ref18] Kumar S. Stecher G. Tamura K. (2016). MEGA7: molecular evolutionary genetics analysis version 7.0 for bigger datasets. Mol. Biol. Evol. 33, 1870–1874. doi: 10.1093/molbev/msw054, PMID: 27004904 PMC8210823

[ref19] Lastochkina O. Seififikalhor M. Aliniaeifard S. Baymiev A. Pusenkova L. (2019). Bacillus spp.: efficient biotic strategy to control postharvest diseases of fruits and vegetables. Plan. Theory 8:97. doi: 10.3390/plants8040097, PMID: 31013814 PMC6524353

[ref20] Leelasuphakul W. Hemmanee P. Chuenchitt S. (2008). Growth inhibitory properties of Bacillus subtilis strains and their metabolites against the green mold pathogen (Penicillium digitatum Sacc.) of citrus fruit. Postharvest Biol. Technol. 48, 113–121. doi: 10.1016/j.postharvbio.2007.09.024

[ref21] Liao J. Jiang W. Y. Wu X. J. He J. Li H. L. Wang T. S. (2022). First report of apiospora mold on sugarcane in China caused by Apiospora arundinis (Arthrinium arundinis). Plant Dis. 106:1058. doi: 10.1094/PDIS-02-21-0386-PDN, PMID: 34546783

[ref22] Liu R. Li J. Zhang F. Zheng D. Chang Y. Xu L.. (2021). Biocontrol activity of Bacillus velezensis D4 against apple Valsa canker. Biol. Control 163:104760. doi: 10.1016/j.biocontrol.2021.104760

[ref23] Liu X. J. Luo X. Y. (1984). Etiological study on poisoning of spoiled sugarcane I – analysis of epidemiological and clinical data and toxicity test of poisoned samples. Health research. 13, 24–28.

[ref24] Liu X. J. Luo X. Y. Li X. F. Chen Q. T. (1988). Classification and identification of the pathogen of spoilage sugarcane poisoning. J. Fungi 7, 221–225.

[ref25] Liu X. J. Luo X. Y. Liu X. M. Li X. F. Li Y. W. (1984). Etiological study on poisoning of spoiled sugarcane II – isolation and toxicity test of pathogenic fungi. Health Res. 13, 28–33.

[ref26] Liu J. Sui Y. Wisniewski M. Droby S. Liu Y. (2013). Review: utilization of antagonistic yeasts to manage postharvest fungal diseases of fruit. Int. J. Food Microbiol. 167, 153–160. doi: 10.1016/j.ijfoodmicro.2013.09.004, PMID: 24135671

[ref27] Liu X. J. Sun Y. Y. Li X. F. Liu J. Wang Y. H. (1993). Studies on the prevention of poisoning of spoiled sugarcane II. Studies on the ecology of the pathogen of spoiled sugarcane poisoning. Hygiene Res. 22, 96–98.

[ref28] Myo E. M. Liu B. Ma J. Shi L. Jiang M. Zhang K. (2019). Evaluation of Bacillus velezensis NKG-2 for bio-control activities against fungal diseases and potential plant growth promotion. Biol. Control 134, 23–31. doi: 10.1016/j.biocontrol.2019.03.017

[ref29] Nagpure A. Choudhary B. Gupta R. K. (2014). Mycolytic enzymes produced by Streptomyces violaceusniger and their role in antagonism towards wood-rotting fungi. J. Basic Microbiol. 54, 397–407. doi: 10.1002/jobm.201200474, PMID: 23686763

[ref30] Niem J. M. Billones-Baaijens R. Stodart B. Savocchia S. (2020). Diversity profiling of grapevine microbial Endosphere and antagonistic potential of Endophytic pseudomonas against grapevine trunk diseases. Front. Microbiol. 11:477. doi: 10.3389/fmicb.2020.00477, PMID: 32273871 PMC7113392

[ref31] Nony P. A. Scalle A. C. T. Rountree R. L. Ye X. Binienda Z. (1999). 3-Nitropropionic acid (3-NPA) produces hypothermia and inhibits histochemical Labeling of succinate dehydrogenase (SDH) in rat brain. Metab. Brain Dis. 14, 83–94. doi: 10.1023/a:1020753629477 10488910

[ref32] Nunes C. Bajji M. Stepien V. Manso T. Torres R. (2008). Development and application of a SCAR marker to monitor and quantify populations of the postharvest biocontrol agent Pantoea agglomerans CPA-2. Postharvest Biol. Technol. 47, 422–428. doi: 10.1016/j.postharvbio.2007.07.016

[ref33] Parafati L. Vitale A. Restuccia C. Cirvilleri G. (2015). Biocontrol ability and action mechanism of food-isolated yeast strains against Botrytis cinerea causing post-harvest bunch rot of table grape. Food Microol. 47, 85–92. doi: 10.1016/j.fm.2014.11.013, PMID: 25583341

[ref34] Rivas-Garcia T. Murillo-Amador B. Nieto-Garibay A. Rincon-Enriquez G. (2019). Enhanced biocontrol of fruit rot on muskmelon by combination treatment with marine Debaryomyces hansenii and Stenotrophomonas rhizophila and their potential modes of action. Postharvest Biol. Technol. 151, 61–67. doi: 10.1016/j.postharvbio.2019.01.013

[ref35] Shakeel M. Rais A. Hassan M. N. Hafeez F. Y. (2015). Root associated bacillus sp improves growth, yield and zinc translocation for basmati rice (Oryza sativa) varieties. Front. Microbiol. 6:1286. doi: 10.3389/fmicb.2015.01286, PMID: 26635754 PMC4649038

[ref36] Souza S. O. Costa S. S. L. Brum B. C. T. Santos S. H. Garcia C. A. B. Araujo R. G. O. (2019). Determination of nutrients in sugarcane juice using slurry sampling and detection by ICP OES. Food Chem. 273, 57–63. doi: 10.1016/j.foodchem.2018.03.060, PMID: 30292375

[ref37] Spadaro D. Droby S. (2016). Development of biocontrol products for postharvest diseases of fruit: the importance of elucidating the mechanisms of action of yeast antagonists. Trends Food Sci Tech. 47, 39–49. doi: 10.1016/j.tifs.2015.11.003

[ref38] Subbanna A. R. N. S. Rajasekhara H. Stanley J. Mishra K. K. Pattanayak A. (2018). Pesticidal prospectives of chitinolytic bacteria in agricultural pest management. Soil Biol. Biochem. 116, 52–66. doi: 10.1016/j.soilbio.2017.09.019

[ref39] Vos P. Garrity G. Jones D. Krieg N. R. Ludwig W. Rainey F. A.. (2011). Bergey’s manual of systematic bacteriology, 3: The Firmicutes. Cham: Springer Science & Business Media

[ref40] Waewthongrak W. Pisuchpen S. Leelasuphakul W. (2015). Effect of Bacillus subtilis and chitosan applications on green mold (Penicilium digitatum Sacc.) decay in citrus fruit. Postharvest Biol. Technol. 99, 44–49. doi: 10.1016/j.postharvbio.2014.07.016

[ref41] Wang K. L. Deng Q. Q. Chen J. W. Shen W. K. (2020). Physiological and molecular mechanisms governing the effect of virus-free chewing cane seedlings on yield and quality. Sci. Rep. 10:10306. doi: 10.1038/s41598-020-67344-4, PMID: 32587358 PMC7316764

[ref42] Wang N. N. Yan X. Gao X. N. Niu H. J. Kang Z. S. Huang L. L. (2016). Purification and characterization of a potential antifungal protein from Bacillus subtilis E1R-J against Valsa Mali. World J Microb Biot. 32, 63–10. doi: 10.1007/s11274-016-2024-5, PMID: 26925625

[ref01] Wei D. L. Chang S. C. Lin S. C. Doong M. L. Jong S. C. (1994). Production of 3-nitropropionic acid by Arthrinium species. Curr. Microbiol. 28, 1–5. doi: 10.1007/bf01575978

[ref43] Xu T. Zhu T. Li S. (2016). β-1, 3-1, 4-glucanase gene from Bacillus velezensis ZJ20 exerts antifungal effect on plant pathogenic fungi. World J. Microb. Biot. 32, 26–3993. doi: 10.1007/s11274-015-1985-0, PMID: 26745986

[ref44] Xue L. Xue Q. Chen Q. Lin C. Shen G. Zhao J. (2013). Isolation and evaluation of rhizosphere actinomycetes with potential application for biocontrol of Verticillium wilt of cotton. Crop Prot. 43, 231–240. doi: 10.1016/j.cropro.2012.10.002

[ref45] Yang Z. Mm L. Sp H. Tang L. Xp C. Tx G. (2023). Inhibitory effect of volatile bacillus on mango anthrax and its preventive effect on mango anthracnose. Acta Phytopathol. Sin. 1312, 1–17. doi: 10.13926/j.cnki.apps.001312

[ref46] Yu X. Ai C. Xin L. Zhou G. (2011). The siderophore-producing bacterium,Bacillus subtilis CAS15, has a biocontrol effect on Fusarium wilt and promotesthe growth of pepper. Eur. J. Soil Biol. 47, 138–145. doi: 10.1016/j.ejsobi.2010.11.001

[ref47] Yuan H. Shi B. Wang L. Huang T. Zhou Z. Hou H. (2022). Isolation and characterization of Bacillus velezensis strain P2-1 for biocontrol of apple postharvest decay caused by Botryosphaeria dothidea. Front. Microbiol. 12:808938. doi: 10.3389/fmicb.2021.808938, PMID: 35058916 PMC8764377

[ref48] Zeriouh H. de Vicente A. Pérez-García A. Romero D. (2014). Surfactin triggers biofilm formation of Bacillus subtilis in melon phylloplane and contributes to the biocontrol activity. Environ. Microbiol. 16, 2196–2211. doi: 10.1111/1462-2920.12271, PMID: 24308294

[ref49] Zhang H. Ma L. Wang L. Jiang S. Dong Y. Zheng X. (2008). Biocontrol of gray mold decay in peach fruit by integration of antagonistic yeast with salicylic acid and their effects on postharvest quality parameters. Biol. Control 47, 60–65. doi: 10.1016/j.biocontrol.2008.06.012

[ref50] Zhang J. Meng L. Zhang Y. Sang L. Wang G. (2020). Gapb is involved in biofilm formation dependent on lrgab but not the sini/r system in bacillus cereus 0-9. Front. Microbiol. 11:591926. doi: 10.3389/fmicb.2020.591926, PMID: 33365021 PMC7750190

[ref51] Zhang S. Zheng Q. Xu B. Liu J. (2019). Identification of the fungal pathogens of postharvest disease on peach fruits and the control mechanisms of Bacillus subtilis JK-14. Toxins 11:322. doi: 10.3390/toxins11060322, PMID: 31195675 PMC6628418

[ref52] Zhou M. Li P. Wu S. Zhao P. Gao H. (2019). Bacillus subtilis cf-3 volatile organic compounds inhibit monilinia fructicola growth in peach fruit. Front. Microbiol. 10:1804. doi: 10.3389/fmicb.2019.01804, PMID: 31440224 PMC6692483

[ref53] Zhou J. Xie Y. Liao Y. Li X. Li Y. Li S. (2022). Characterization of a Bacillus velezensis strain isolated from Bolbostemmatis Rhizoma displaying strong antagonistic activities against a variety of rice pathogens. Front. Microbiol. 13:983781. doi: 10.3389/fmicb.2022.983781, PMID: 36246295 PMC9555170

