# Supplementary figures and images for: Biocontrol ability of Bacillus velezensis T9 against Apiospora arundinis causing Apiospora mold on sugarcane
Source: Front Microbiol. 2023 Dec 22;14:1314887. doi: 10.3389/fmicb.2023.1314887 (PMC10766759; doi:10.3389/fmicb.2023.1314887)

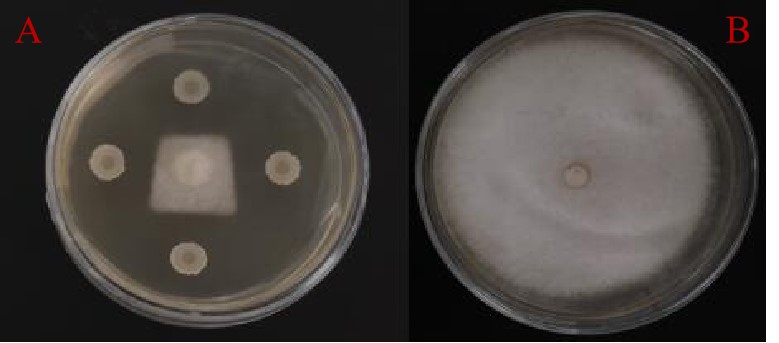

Supplement: SUPPLEMENTARY FIGURE S1 — Antifungal activity of strain T9 against A. arundinis. (A) Antifungal activity of strain T9 against A. arundinis. (B) untreated A. arundinis. [file Image_1.JPEG]

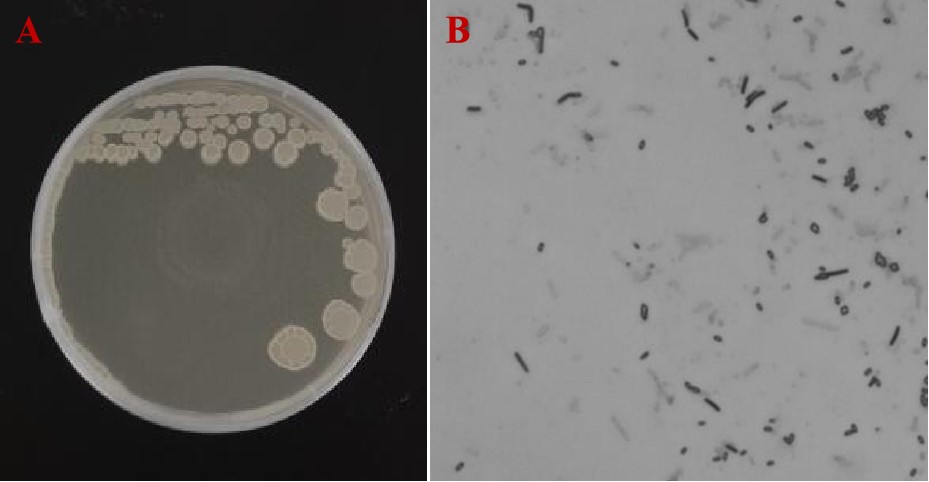

Supplement: SUPPLEMENTARY FIGURE S2 — Colony morphological characteristics of the strain T9. (A) Strain T9 colony morphology. (B) Gram staining of thestrain T9. [file Image_2.JPEG]

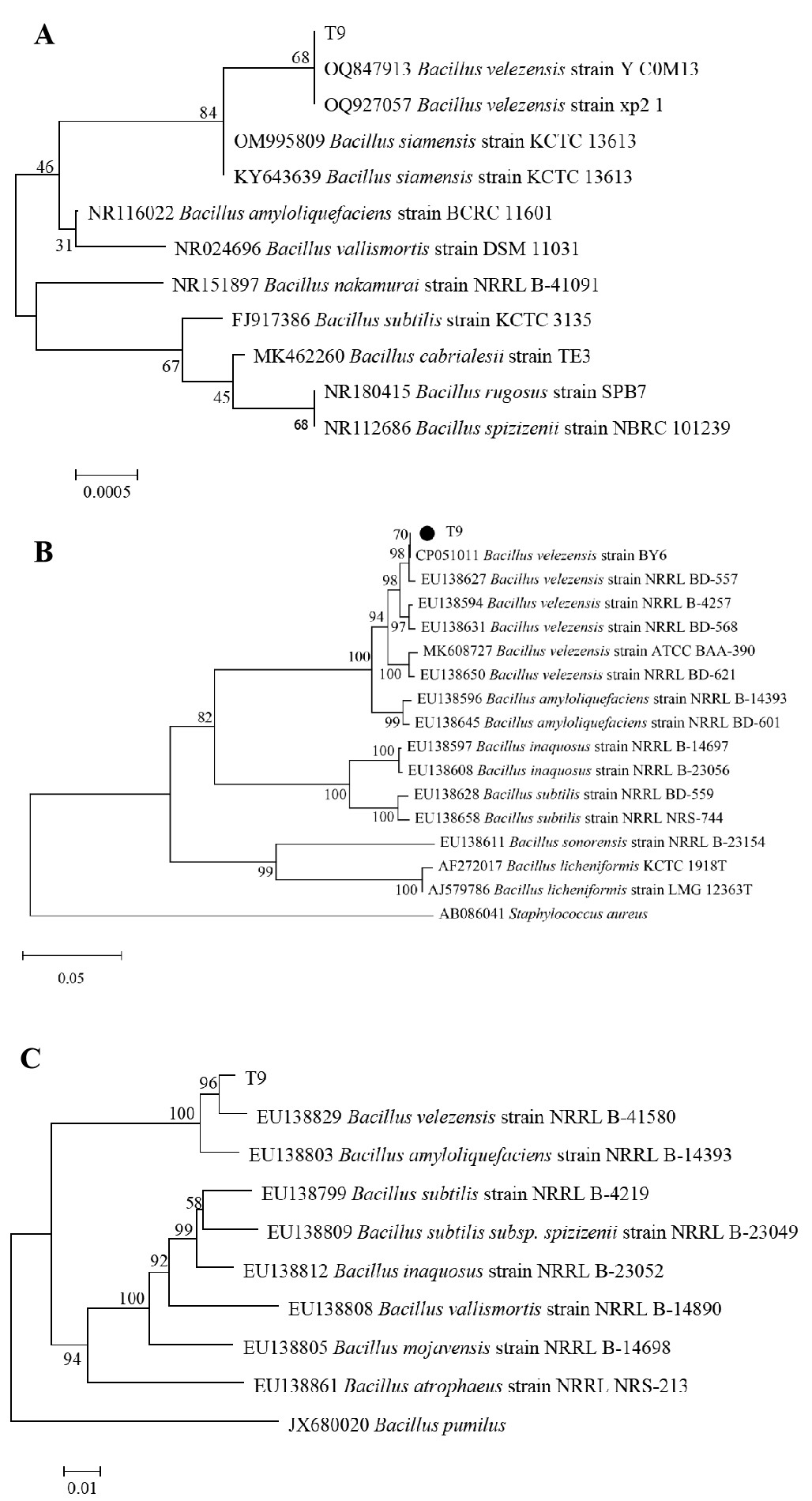

Supplement: SUPPLEMENTARY FIGURE S3 — Neighbor-joining phylogenetic tree of the strain T9. Neighbor-joining phylogenetic tree of the strain T9 were constructed by MEGA7.0 basing on 16 S rDNA (A), gyrA (B), and ropB (C). The numbers at the branches indicate the confifidence level calculated by bootstrap analysis (1000). [file Image_3.JPEG]
