# Supplementary material for: Biocontrol ability of Bacillus velezensis T9 against Apiospora arundinis causing Apiospora mold on sugarcane
Source: Front Microbiol. 2023 Dec 22;14:1314887. doi: 10.3389/fmicb.2023.1314887 (PMC10766759; doi:10.3389/fmicb.2023.1314887)
Supplement: Supplementary file 4 [file Table_1.DOCX]

**Supplementary Table legend**

Supplementary Table 1. Part of physiological and biochemical characters of strain T9

| Characteristic index | Result | Characteristic index | Result |
| --- | --- | --- | --- |
| Gram stain | + | pH 5 | + |
| Voges-Proskauer (VP) test | + | pH 7 | + |
| Citrate | - | pH 8 | + |
| Methyl red test | + | pH 9 | + |
| gelatin liquefaction | + | 20℃ | + |
| Starch hydrolysis | + | 30℃ | + |
| glucose | + | 40℃ | + |
| D-sorbitol | + | 50℃ | + |
| D-fructose | + | 2% NaCl | + |
| D-mannitol | + | 5% NaCl | + |
| proline | + | 7% NaCl | + |
| alanine | + | 10% NaCl | - |
|  |  |  |  |

+ and − represent positive and negative reactions, respectively.

Data within each column are the means of three independent experiments.
